# Crosstalk Between *Cis*-Regulatory Elements and Metabolism Reprogramming in Hepatocellular Carcinoma

**DOI:** 10.3390/cancers18061002

**Published:** 2026-03-19

**Authors:** Yuqing Ren, Di Tang, Xiaofan Ding, Mian He

**Affiliations:** 1 Faculty of Health Sciences, University of Macau, Macau SAR 999078, China; yc37655@um.edu.mo (Y.R.); dingxiaofan@um.edu.mo (X.D.); 2 Department of General Surgery, The Seventh Affiliated Hospital of Sun Yat-sen University, Shenzhen 518107, China; tangdi@mail.sysu.edu.cn; 3 MOE Frontiers Science Center for Precision Oncology, University of Macau, Macau SAR 999078, China

**Keywords:** *Cis*-regulatory elements, hepatocellular carcinoma, metabolic reprogramming

## Abstract

Hepatocellular carcinoma (HCC) is a leading cause of cancer-related deaths worldwide, arising from profound metabolic reprogramming and widespread epigenetic dysregulation. However, the role of epigenetic aberrations in modulating metabolic reprogramming and the interplay between *cis*-regulatory elements (CREs), such as promoters, enhancers and super-enhancers, and metabolic adaptation have not been systematically summarized. Therefore, this review aims to integrate current evidence to elucidate the mechanisms of how *cis*-regulatory elements (CREs) drive oncogenic and metabolic signals in HCC progression. For instance, enhancers and super-enhancers transcriptionally activate key metabolic genes involved in aerobic glycolysis (*GLUT1*, *HK2*, *PKM2*, *LDHA*), de novo lipogenesis (*ACLY*, *FASN*, *ACC*), glutaminolysis (*SLC1A5*, *GLS*), and nucleotide synthesis. Meanwhile, many metabolic intermediates, including acetyl-CoA, succinyl-CoA and lactate, act as cofactors or substrates for epigenetic modifiers, creating bidirectional feedback loops that reinforce CRE-driven malignant phenotypes. Therefore, aberrant CREs acts as “metabolic switches” that sense and respond to various metabolic conditions to sustain HCC growth. Consequently, targeted intervention against oncogenic CREs, such as super-enhancers or their co-activators, to disrupt CRE-mediated metabolic vulnerabilities, has emerged as a highly promising new paradigm for precision therapy in HCC.

## Simple Summary

Metabolic reprogramming is a fundamental hallmark of hepatocellular carcinoma (HCC), in which *cis*-regulatory elements (CREs) play a pivotal role. This review provides a systematic overview of the definition, identification, and biological functions of CREs in HCC-associated metabolic reprogramming. Aberrant CREs contribute substantially to tumorigenesis through multiple mechanisms, including promoter hypermethylation, enhancer hijacking and disruption of 3D chromatin organization. Furthermore, HCC progression is complicated by the bidirectional crosstalk between CREs and metabolic reprogramming. CREs orchestrate the transcription of core metabolic enzymes, while metabolic intermediates reciprocally fine-tune CRE activity by acting as substrates or cofactors for chromatin-modifying enzymes. Considering this interplay, novel therapeutic strategies aimed at targeting key oncogenic CREs may exploit CRE-metabolism vulnerabilities for better interventions in HCC.

## 1. Introduction

Hepatocellular carcinoma (HCC), the most common primary liver malignancy, is a leading cause of cancer-related mortality worldwide [1]. Its rising incidence is driven largely by the prevalence of chronic liver diseases, including hepatitis B and C infections, alcohol-related liver disease, and the growing burden of non-alcoholic fatty liver disease (NAFLD) and non-alcoholic steatohepatitis (NASH) [2,3]. Despite improvements in surveillance and treatment, HCC prognosis remains poor, with a 5-year overall survival rate below 30% [3,4]. The dismal outlook stems primarily from late diagnosis, frequent recurrence, and the limited efficacy of current systemic therapies [5].

Epigenetic dysregulation is a key driver of hepatocarcinogenesis, rewiring transcriptional programs through alterations in DNA methylation, histone modifications, and three-dimensional (3D) genome organization [6,7,8]. Central to this process is the reprogramming of *cis*-regulatory element (CRE) landscapes. Activation of enhancers or acquisition of super-enhancers at critical genomic loci hijacks normal regulatory circuits to promote malignancy and metabolic adaptation, which underpins the development of epigenetic therapies for HCC.

Metabolic reprogramming in HCC is characterized by enhanced glycolysis, altered tricarboxylic acid (TCA) cycle flux, upregulated lipid/cholesterol synthesis, rewired amino acid utilization, and suppression of urea cycle function [9,10]. Although traditionally attributed to oncogenic signaling, hypoxia, or mitochondrial dysfunction, these metabolic phenotypes are now understood to be, at least in part, actively instructed by chromatin-level regulation [11,12]. The reprogramming of CREs fuels the sustained overexpression of metabolic genes, thereby conferring a selective advantage that drives tumor growth and adaptation [13,14,15,16].

The interplay between CREs and metabolism HCC is fundamentally bidirectional. Metabolic intermediates such as acetyl-CoA, S-adenosylmethionine (SAM), α-ketoglutarate (α-KG), and nicotinamide adenine dinucleotide (NAD^+^) act as essential cofactors for chromatin-modifying enzymes, directly coupling cellular metabolic states to the epigenetic landscape [17,18,19,20,21,22]. This metabolic–epigenetic feedback reshapes oncogenic CRE activity, establishing a self-reinforcing loop that stabilizes malignant transcriptional programs. Furthermore, 3D genome organization and the tumor microenvironment add further complexity into this regulatory network [23,24,25].

While extensive research has documented the landscape of epigenetic and metabolic alterations in HCC, the fundamental mechanism by which CREs orchestrate metabolic reprogramming remains a critical and unresolved challenge. In this review, we synthesize recent advances in understanding the mechanistic links between CRE reprogramming and metabolic rewiring in HCC. We first outline the altered landscape of CREs, encompassing enhancer activity and 3D genome architecture, and summarize key dysregulated metabolic pathways. We then explore the bidirectional crosstalk between CREs and metabolic reprogramming and their therapeutic implications. By integrating epigenetic and metabolic perspectives, this review aims to provide a conceptual framework for understanding HCC pathogenesis and to highlight novel vulnerabilities for precision therapy.

## 2. The *Cis*-Regulatory Elements in Hepatocellular Carcinoma

**Figure 1:**
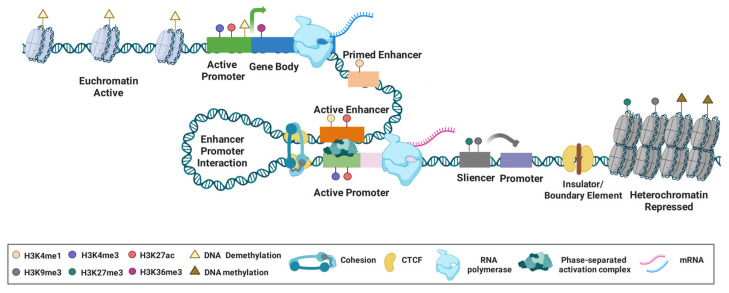
Schematic diagram of CREs and their epigenomic marks. This diagram delineates the core components of CREs and the epigenomic signatures that demarcate their activity states in eukaryotic genomes. Promoters are typically located proximal to TSSs and are characterized by high levels of H3K4me3 and H3K27ac, frequent association with hypomethylated CpG islands, and occupancy by RNAP II. Enhancers are distal regulatory sequences that activate target genes via long-range interactions. Active enhancers are commonly marked by enrichment of H3K4me1, H3K27ac, p300/CBP binding, and open chromatin, whereas primed enhancers are primarily enriched for H3K4me1. Silencers are repressive distal elements, often associated with H3K27me3, H3K9me3, or occupancy by PRC, and exhibit low or absent histone acetylation. Insulators function as boundary elements that block enhancer–promoter communication or prevent the spread of heterochromatin. They are frequently bound by CTCF and/or cohesin and are characterized by specific chromatin looping patterns associated with CTCF motifs. The diagram further illustrates how the combinatorial patterns of these histone modifications, chromatin accessibility, DNA methylation, and 3D chromatin architecture such as enhancer–promoter looping collectively constitute the regulatory landscape and govern cell-type-specific gene expression.

Gene expression is initiated when RNA polymerases are recruited to transcriptional start sites (TSSs), enabling RNA synthesis from a DNA template. This process is regulated at higher order through the binding of DNA-binding proteins, particularly transcription factors (TFs), to CREs. As the fundamental regulatory units of the epigenome, CREs are non-coding genomic regions that direct context- and cell-type-specific gene transcription [26,27,28]. These elements include promoters, enhancers, silencers, insulators, and chromatin interaction anchors, which collectively integrate transcription factor binding, chromatin modifications, and higher-order genome organization to orchestrate precise transcriptional programs [29,30] (Figure 1). Advances in genomic resources and computational tools, such as those from ENCODE Phase IV (ENCODE4), have enabled the systematic identification of 2.37 million human and 967,000 mouse CREs [31]. In eukaryotic genomes, tens of thousands of CREs encode combinatorial logic that dictates when, where, and to what level a gene is expressed [32].

### 2.1. Definition and Identification of CREs

CREs can be systematically identified through direct and indirect methods. Direct approaches detect DNA sequences physically bound by TFs, while indirect approaches locate CREs based on related epigenetic features such as chromatin accessibility, histone modification, or transcription activation that follows TF binding.

Promoters: Promoters are *cis*-regulatory elements positioned at or near TSSs and serve as the primary element in transcription initiation. Active promoters are often characterized by a distinct chromatin architecture, including nucleosome depletion, H3K4me3 enrichment and assembly of the basal transcriptional machinery, facilitating the recruitment of RNA polymerase II (RNAP II) and the onset of transcription [33]. There are an estimated 10,567 active promoters in the human genome [34], typically ranging from 100 to 1000 base pairs in length [33]. The structure of a promoter comprises a core promoter and may include proximal regulatory sequences. The core promoter, spanning the region surrounding the TSS, contains conserved DNA elements such as TATA box that facilitate the assembly of the pre-initiation complex and ensure accurate transcription initiation [35]. Adjacent to the core promoter, the proximal promoter harbors binding sites for sequence-specific transcription factors, which modulate transcriptional activity in response to various physiological or environmental signals. Active promoters are characterized by specific histone marks, most notably H3K4me3 and H3K27ac, the latter of which creates an open chromatin structure permissive for transcription. Repressed promoters are often marked by H3K9me3 or H3K27me3. Additionally, hypermethylation of CpG islands within promoter regions is a well-established epigenetic hallmark in cancers, leading to transcription silencing of tumor suppressor genes (TSGs) and contributing to oncogenesis [36].

Enhancers: Enhancers are regulatory elements that potently stimulate transcription from a distance, independent of their orientation or precise location relative to the target gene [37,38]. They were first identified in Simian virus 40 genome as a cluster of short DNA sequences capable of boosting the transcription efficiency of target genes [39]. The activity and regulatory potential of these elements are defined by complex histone modification patterns extending beyond canonical marks like H3K4me1 and H3K27ac [40] to include diverse acetylation patterns such as H3K9ac and H4K16ac, as well as non-acetyl modifications like crotonylation, lactylation and β-hydroxybutyrate at sites such as H3K56 [41,42,43,44,45]. In parallel, enhancers can be identified through the enrichment of specific transcription factors and co-regulators, such as BRD4, C/EBPα, p300, MED1, and lineage-defining factors like MyoD, T-bet, Oct4, Sox2, Nanog and PU.1 [46,47,48,49]. Based on their genomic and epigenetic features, enhancers are broadly classified into typical enhancers (TEs) and super-enhancers (SEs). Typical enhancers (TEs) function individually or in loose clusters, often regulating genes from upstream or downstream positions [50]. Super-enhancers (SEs) are large, contiguous clusters of enhancer elements, usually within 12.5 kb, marked by exceptionally high occupancy of the mediator co-activator complex and elevated levels of histone modifications such as H3K27ac and H3K4me1 [51,52]. In HCC, the enhancer and super-enhancer landscape is extensively reprogrammed, which plays a critical role in driving the expression of genes involved in cancer progression, metabolism reprogramming and other malignant phenotypes [46,53,54].

Silencers: Silencers are regulatory DNA elements that repress gene transcription by binding repressive transcription factors or recruiting chromatin-modifying complexes [55]. Typically located distally from their target promoters, often thousands of base pairs away, they function in a cell-type-specific manner to fine-tune gene expression during development, differentiation, and lineage specification. Currently, there is no universally accepted method for identifying silencers. Huang et al. proposed an approach based on the correlation between H3K27me3-marked DNase I hypersensitive site (DHS) and gene expression [56]. Genomic regions enriched in H3K27me3 can indeed act as silencers to repress transcription through chromatin interactions [57]. Genome-wide studies have further revealed over 1.7 million candidate silencers across diverse human cell types [55]. Silencers inhibit transcription through multiple mechanisms, including disrupting enhancer–promoter interactions via altered chromatin looping, competing with activators for DNA binding sites, and recruiting repressive complexes such as Polycomb repressive complex 2 (PRC2) to deposit repressive histone marks like H3K27me3 [57]. Beyond their role in gene regulation, silencers are significantly enriched for expression quantitative trait loci (eQTLs) and disease-associated single nucleotide polymorphisms (SNPs) [58].

Insulators: Insulators are specialized *cis*-regulatory elements, typically spanning 300 to 2000 base pairs, that act as genomic barriers to organize chromatin architecture and precisely regulate gene expression. The functions of insulators include enhancer blocking, which prevents enhancers from activating inappropriate promoters, and boundary function, which restricts the spread of repressive chromatin marks such as H3K27me3 into active domain and vice versa [59]. Through these dual roles, insulators maintain genomic compartmentalization, ensuring proper transcriptional control and preventing aberrant gene activation or silencing. Insulators function mainly by binding specific insulator binding proteins (IBPs), among which the CCCTC-binding factor (CTCF) is the most well-characterized and evolutionarily conserved zinc-finger protein [60]. CTCF plays a pivotal role in chromatin organization and transcriptional regulation by facilitating the formation of topologically associating domains (TADs) and chromatin loops [61]. This structural organization ensures appropriate enhancer–promoter interactions while preventing aberrant regulatory crosstalk [62].

### 2.2. The Role of CREs in HCC

CREs orchestrate gene expression by modulating chromatin accessibility and the three-dimensional genome architecture. In HCC, aberrant activity of these elements can lead to inappropriate activation of oncogenes and repression of tumor suppressor genes. Such epigenetic reprogramming contributes to key malignant phenotypes, such as uncontrolled proliferation and metastasis, positioning CREs as central players in HCC pathogenesis.

#### 2.2.1. Promoter Methylation Is Closely Associated with HCC Progression

As core regulatory elements, promoters initiate transcription through the recruitment of RNA polymerase by sequence specific transcription factors. Their activity is tightly regulated by epigenetic modifications. For instance, cytosine methylation at CpG islands in the promoter region typically impedes transcription factor binding and silences gene expression [63]. In cancer, promoters frequently undergo dysregulation through hypermethylation, leading to the silencing of tumor suppressor genes and facilitating tumor development.

In HCC, promoter dysregulation is widespread and functionally consequential. Genome-wide analyses have identified 855 alternative promoters whose activity strongly correlated with DNA methylation status [64]. Furthermore, promoters and enhancers exhibit coordinated dynamics in chromatin accessibility and regulatory activity during DNA damage responses, suggesting integrated transcriptional rewiring in HCC progression [65]. Specific promoter alterations have been linked to clinical and functional outcomes. For instance, hypermethylation of the *RASSF1A* and *DOK1* promoters serves as a potential biomarker for early HCC detection and a target for epigenetic therapy [66]. Similarly, *PAX6* promoter hypermethylation downregulates its expression, promoting tumor growth and metastasis via regulation of *CDH1* and *THBS1* [67]. FOXC1 upregulates *DNMT3B* to induce hypermethylation and silencing of *CTH*, driving HCC proliferation through a ROS-mediated feedback loop [68]. Sequential DNA methylation alterations during hepatocarcinogenesis also lead to *ZNF334* silencing via disrupting *TP53* promoter binding, further accelerating HCC progression [69]. Additionally, hypomethylation of the fetal promoter of *IGF2* reactivates this oncogene in HCC [70].

The functional study of promoters relies on multi-level experimental strategies. Omics level approaches such as CAGE-seq enable genome-wide promoter identification, including novel alternative promoters outside CpG islands that are implicated in hepatocarcinogenesis [71]. For experimental validation, classical reporter assays offer a robust and quantitative approach to monitor promoter activity, making them suitable for functional validation and for assessing the effects of specific mutations, while Massively Parallel Reporter Assays (MPRAs) can provide quantitative comparison of the activities of thousands of promoters in parallel [72]. When integrated with complementary techniques such as mutational analysis, electrophoretic mobility shift assays (EMSAs), and DNase I footprinting, these assays allow for the precise mapping of essential promoter regions and the systematic evaluation of how individual mutations alter transcription regulation [73].

#### 2.2.2. The Oncogenic Role of Enhancers in HCC

Enhancers are essential epigenetic regulators that play critical roles in cell development and disease progression. In HCC, large-scale integrative analyses have established a tight link between enhancer dysregulation and tumorigenesis. For instance, Yang et al. demonstrate that de novo enhancers emerging in cirrhosis and conserved in HCC drive tumorigenesis and evolution by coordinately regulating hepatocyte-intrinsic pathways and tumor immune responses [74]. Multi-omics epigenetic analyses further reveal coordinated disruptions in DNA hydroxymethylation, methylation and histone modifications particularly through enhancer remodeling [75]. Accumulating evidence underscores enhancers as pivotal regulators of key oncogenic processes in HCC, including proliferation, migration, invasion, and epithelial–mesenchymal transition (EMT).

During hepatocarcinogenesis, enhancers and super-enhancers (SEs) drive the expression of central oncogenes. Tumor-specific enhancer activation directly leads to aberrant overexpression of canonical oncogenes such as *MYC*, *HSPA4*, *KLF5* and *SPHK1*, thereby reinforcing malignant phenotypes of HCC [46,76,77,78]. Integration of hepatitis B virus (HBV) DNA can lead to *cis*-activation of nearby oncogenes, contributing to accelerated disease progression in HCC [79]. Moreover, transposable elements (TEs) can act as *cis*-regulatory elements by attracting transcription factors and epigenetic regulators, thereby influencing oncogenic gene expression networks [80]. This SE-mediated transcriptional activation establishes a state of oncogenic addiction, rendering HCC cells highly dependent on these regulatory elements for survival and proliferation.

Carcinogenesis is a highly intricate process that involves not only the dysregulation of oncogenes but also the functional perturbation of TSGs. In this context, enhancers can modulate TSG expression through epigenetic mechanisms. For instance, the loss of *IGFBP4* in HCC is associated with aberrant signaling transduction, highlighting the role of enhancers in regulating TSG expression [81]. Furthermore, in HCC cells, the *SIRT7* super-enhancer (*SIRT7*-SE) drives its own transcription by facilitating the co-occupancy of C/EBPβ and BRD4, subsequently inducing genome-wide H3K18 deacetylation, and physically interacts with EZH2 to promote cooperative epigenetic silencing, thereby establishing a self-reinforcing oncogenic circuit [15].

Enhancer reprogramming is pivotal in HCC pathogenesis. Comprehensive epigenomic studies have further identified cancer-lineage-specific active enhancers (CL-HCC AEs) as critical determinants of HCC tumorigenesis and evolutionary trajectories, providing a molecular basis for subclassifying HCC and informing precision therapeutics [74]. Systematic mapping of epigenetic alterations across HCC progression has identified recurrent driver events associated with enhancer deregulation that influence tumor initiation and progression [75]. Similarly, distinct genomic enhancer signatures have been shown to stratify HCC patients with different prognostic outcomes [54].

#### 2.2.3. Disruption of Silencers and Insulators in HCC

Silencers are DNA sequences that suppress transcription by recruiting transcriptional repressors, such as the Polycomb repressive complex (PRC), which deposit repressive histone marks like H3K27me3 [82]. In cancer, disruption of silencer function is common and can lead to oncogene activation. For example, loss of H3K27me3 has been linked to the derepression of oncogenes *MYH11* and *EGFR*, contributing to tumor progression [83]. In HCC, overexpression of EZH2, the catalytic subunit of PRC2, leads to aberrant accumulation of H3K27me3 at promoters and distal regulatory elements of TSGs (e.g., *P21*, *CHD5*), thereby inactivating their expressions [84,85]. Similarly, SE promotes the transcriptional silencing of TSGs by inducing genome-wide deacetylation of H3K18 and cooperatively interacting with EZH2 to reinforce H3K27me3-mediated repression [15]. This silencing affects key regulators such as *EGR2*, *IRF8*, *SOCS*, and *ZBTB*, impairing metabolic and immune regulatory functions and enhancing tumorigenicity in HCC [15].

Insulators, primary mediated by the architectural protein CTCF, help maintain the three-dimensional topological structure of the genome and delineate transcription domains. There are over 50,000 CTCF binding sites in the genome and CTCF binding can be modulated by DNA methylation within its recognition sequence [86]. Disruption of CTCF function is frequently observed in cancer [87]. For example, hypermethylation of the *CDKN2A* promoter prevents CTCF binding at a nearby chromatin boundary, leading to loss of insulation, chromatin compaction and transcription silencing of this key tumor suppressor [88]. In HCC, CTCF expression is frequently upregulated, and elevated CTCF levels are associated with poorer patient prognosis [89]. Moreover, liver-specific deletion of CTCF in experimental models leads to hepatic steatosis. This phenotype is driven by enhanced PPARγ DNA-binding activity, which upregulates downstream target genes involved in lipid metabolism [90]. These findings highlight the critical role of dysregulated CTCF insulators in promoting HCC progression.

#### 2.2.4. Three-Dimensional Genome Disorganization and Chromatin Dynamics in HCC

Accumulating evidence underscores that cell-type-specific transcriptional programs depend not only on the linear genomic sequence but also on the 3D genome architecture, which facilitates physical interactions between CREs and their target promoters through chromatin looping mediated by TFs and architectural proteins [23,87,88,89,90]. The 3D genome is organized into hierarchical and dynamic frameworks, including active/inactive transcription (A/B compartments), topologically associating domains (TADs) that insulate genomic regions (~100 kb–1 Mb), and specific chromatin loops that bring enhancers into proximity with promoters [91]. These structures are largely maintained by architectural proteins such as CTCF and cohesin, which mediate loop extrusion and contribute to precise gene regulation.

Higher-order genome disorganization is a hallmark of the transition from normal to neoplastic states, observed across diverse cancers [92,93,94,95]. Disruption of the 3D epigenome plays a central and driving role in HCC. For instance, Feng et al. compared the 3D epigenomes of THLE-2 and HepG2 cells and revealed that most HCC-associated genes are organized within complex chromatin interactions mediated by RNAP II [23]. This structural reorganization involves extensive enhancer reprogramming and chromatin loop remodeling, characterized by HCC-specific gains in H3K27ac and aberrant increases in enhancer–promoter looping, which collectively drives the dysregulated activation of oncogenes such as *SOX4* and *GPC3* [54]. Pan-cancer HiChIP analysis of TCGA data further showed tissue-specific *MYC* enhancer biases in liver cancer, suggesting that structural variants can induce differential chromatin looping to activate proto-oncogenes [96]. Moreover, the nuclear matrix protein HNRNPU has been shown to maintain topologically associating domain boundaries essential for preserving 3D genome integrity in hepatocytes [97]. Disruption of this precise spatial architecture thus triggers widespread transcriptional dysregulation, contributing to hepatocyte transformation and HCC progression.

Transcription factors and chromatin remodelers are increasingly recognized as key regulators of 3D genome organization. The Switch/Sucrose non-fermentable (SWI/SNF) chromatin complex, which is mutated in approximately 20% of cancers, critically modulates local enhancer activity [98]. *ARID1A* deficiency alters chromatin conformation and dysregulates genes such as *PMP22* and *GSC*, promoting invasion capacity of liver cancer cells [99]. Similarly, phosphorylation of STAT3 correlates with changes in 3D chromatin architecture that enhance the expression of oncogenes linked to HCC progression and drug resistance [100]. These findings underscore how dynamic chromatin remodeling sustains oncogenic signaling, in part through rewired enhancer–promoter communication.

### 2.3. Somatic Mutations Alter CRE Activity in Cancer

Somatic alterations play a crucial role in cancer initiation and progression. While most cancer genomic studies have focused on coding genes, advances from projects like ENCODE and the reduced cost of whole-genome sequencing (WGS) have revealed the significant role of non-coding somatic mutations [101,102]. It is estimated that approximately 35% of somatic mutations are found in regulatory regions, which may influence cancer development indirectly by disrupting CREs such as promoters and enhancers, thereby affecting gene expression [103]. In HCC, recurrent non-coding mutations have been found in promoters and CTCF binding sites, highlighting their role in carcinogenesis [104].

Promoter mutations can lead to the activation or silencing of genes critical to cellular processes. In HCC, *TERT* promoter mutations are the most common genetic alterations occurring in 44 to 65% of patients, disrupting transcriptional regulation, ultimately contributing to oncogenesis [105,106,107]. These mutations create de novo binding sites for the E26 transformation-specific (ETS) transcription factors, which upregulates *TERT* expression and promotes cellular immortality [108,109]. Furthermore, *TERT* promoter mutations predict primary resistance (PD) to first-line treatments, especially in patients receiving immunotherapy-containing regimens in HCC [110]. Beyond HCC, recurrent promoter mutations in the *SDHD* gene disrupt consensus ETS transcription factor binding sites, reduce *SDHD* gene expression, and are correlated with poor prognosis in melanoma [111]. Similarly, analysis of a large cohort of breast cancer patients identified a mutational hotspot in the *FOXA1* promoter that leads to its overexpression through enhanced E2F binding [112].

In cancer, somatic mutations in enhancers can disrupt TF binding and subsequently alter target gene expression [113]. Small genomic insertions can also create de novo enhancers that drive oncogene expression [114]. For instance, in T-ALL, somatic mutations generated novel MYB binding sites, recruiting transcriptional co-activators such as CBP to drive *TAL1* expression, illustrating how non-coding alterations rewire enhancer activity in cancer [115]. Additionally, somatic SE duplications and hotspot mutations can lead to oncogenic activation of *KLF5* [116]. Structural variants (SVs) such as deletions, inversions, and duplications can disrupt CTCF/cohesin-mediated TAD boundaries, promoting enhancer hijacking. In this process, enhancers or SEs aberrantly activate oncogene expressions. In leukemia, chromosomal rearrangements can reposition an enhancer to simultaneously drive oncogenic *EVI1* expression and cause *GATA2* haploinsufficiency, highlighting a key mechanism of structural-variation-induced enhancer hijacking in cancer pathogenesis [117]. Similarly, promiscuous rearrangements of the MYC locus near super-enhancers (e.g., *IGH, NSMCE2*) lead to its monoallelic overexpression and supporting tumor progression in multiple myeloma [118]. In HCC, hepatitis B virus (HBV) DNA insertions, particularly those carrying viral enhancer elements, into the host genome are well-established drivers of hepatocarcinogenesis [119].

Frequent mutations, deletions, or abnormal DNA methylation at CTCF binding sites leads to widespread loss of insulator function and the collapse of TAD boundaries. Indeed, CTCF/cohesin binding sites are highly mutated across various cancers [120,121,122,123]. An analysis of 1962 whole cancer genomes identified 21 recurrently mutated insulators that likely act as non-coding drivers by rewiring chromatin loop and altering gene expression [124]. Similarly, in liver cancers, four significant mutation clusters have been mapped to CTCF binding regions on chromosomes 2, 3, 18, and 20 [104]. Loss of CTCF binding at the p16 tumor suppressor locus has been correlated with its epigenetic silencing across multiple cancers, promoting unrestricted cell proliferation [88]. In gliomas, loss of CTCF-dependent insulation leads to *MYC* upregulation, as CTCF deletion disrupts the enhancer–promoter looping required for its proper regulation [125]. By weakening genomic insulation, these changes can facilitate aberrant enhancer–promoter interactions, enabling enhancers from one region to inappropriately activate oncogenes elsewhere and promoting malignant gene expression programs.

### 2.4. Defining and Validating CREs Through Integrated Multi-Omics

**Figure 2:**
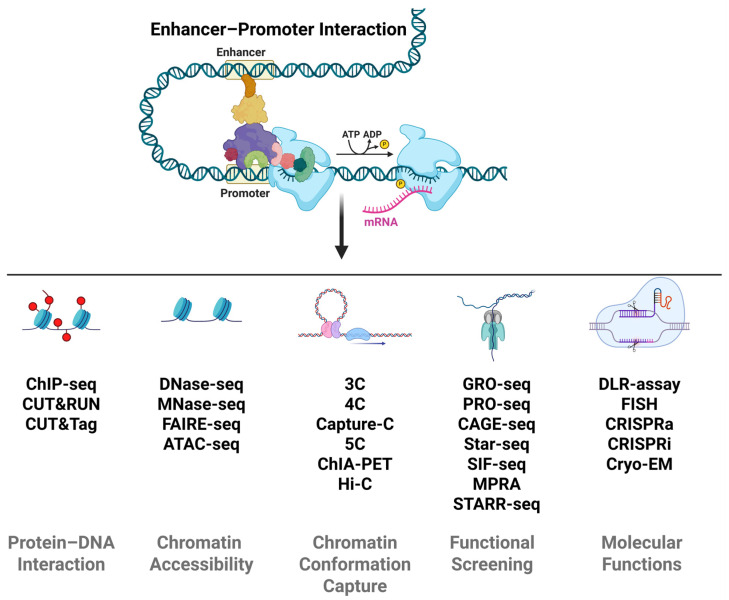
Overview of techniques for studying *cis*-regulatory elements. This diagram summarizes major methodological categories for CRE analysis. Protein–DNA interaction mapping determines the genomic binding sites of transcription factors, cofactors, and histone modifications. Common approaches are ChIP-seq, CUT&RUN, and CUT&Tag. Chromatin accessibility profiling identifies open chromatin regions indicative of active or poised regulatory elements. Key techniques include DNase-seq, MNase-seq, FAIRE-seq, and ATAC-seq. Chromatin conformation capture reveals long-range spatial interactions that mediate regulatory communication. This category includes 3C and its derivatives (4C, 5C, Capture-C, Hi-C) and ChIA-PET. Functional screening assays enable high-throughput testing and direct measurement of enhancer or promoter activity. Representative methods comprise GRO-seq/PRO-seq, CAGE-seq, STARR-seq, SIF-seq, and MPRA. Molecular and functional assays provide targeted perturbation, visualization, or structural insights into CRE mechanisms. Examples are DLR assays, FISH, CRISPRa/i, and Cryo-EM.

Recent advances in multi-omics technologies have provided powerful tools for the systematic identification and functional validation of CREs (Figure 2). By integrating data from genomics, epigenomics, transcriptomics, and 3D genomics, researchers can now comprehensively map CRE landscapes across diverse species, tissues, and pathological conditions. Large-scale epigenome mapping projects, including ENCODE, ROADMAP and 4D-Nucleome, have established standardized analytic frameworks, generating vast public datasets that severe as foundational maps of mammalian CREs and are indispensable for deconstructing complex gene regulatory networks [32,126,127].

Histone modifications and chromatin accessibility serve as core epigenetic signatures for annotating CREs. Techniques such as ChIP-seq, CUT&RUN, and CUT&Tag are widely used to map histone modifications, enabling precise identification of active, poised, or repressed CREs. Chromatin accessibility mapping, including DNase-seq, MNase-seq, FAIRE-seq and ATAC-seq, allows researchers to explore the regions of open chromatin that are accessible to transcription factors and other regulatory proteins [128,129]. By integrating combinatorial patterns of histone markers and chromatin accessibility, ChromHMM can systematically enabling large-scale annotation of putative CREs [130]. Single-cell multi-omics epigenomic techniques could further advanced CRE annotation by overcoming the limitations of bulk analysis [131]. For instance, single-cell ATAC-seq (scATAC-seq) profiles open chromatin regions genome-wide, identifying candidate CREs as accessible peaks [132]. Dedicated computational tools, including SnapATAC [133], ArchR [134], Signac [135], and CREscendo [136], have been developed to enhance precision of peak calling and cell-type-specific CRE decomposition.

The regulatory function of CREs is tightly linked to 3D genome organization. Chromatin conformation capture techniques, including 3C, 4C, Capture-C, 5C, ChIA-PET, and Hi-C, allow for high-throughput mapping of CREs and their interactions in 3D, thus providing insights into spatial organization and functional interaction between CREs [137,138,139,140]. To validating the functional roles of identified CREs, functional screening methods like GRO-seq/PRO-seq, CAGE-seq, STARR-seq, SIF-seq, and Massively Parallel Reporter Assays (MPRAs) enable testing of transcriptional activity for thousands to millions of DNA sequences in vitro, providing quantitative insights into regulatory grammar and the functional impact of genetic variants [141,142,143,144,145,146].

Comprehensive understanding of CRE logic requires the integration of multi-layered data encompassing static epigenetic marks, dynamic chromatin accessibility, and spatial conformation. Many databases for CREs have been bult, such as ENCODE [147], ChIP-Atlas [148], SEdb v2.0 [149], SEA v3.0 [150], dbSUPER [151], and TSCRE [152]. While these databases are invaluable resources, they predominantly profile CREs in normal tissues and cell lines. This focus limits their direct applicability to complex diseases like cancer, where CRE activity is often rewired. There is thus a pressing need for more integrative resources that catalog dysregulated CREs specific to cancer types and states.

Beyond computational methods for mapping and annotating CREs, methodological advances enable large-scale molecular functional validation of these elements. The Dual-Luciferase Reporter assay (DLR assay) is a widely used, sensitive, and quantitative technique to study the activity of CREs [153,154]. Fluorescence in situ hybridization (FISH)-based approaches, particularly Flow-FISH and its variant HCR-FlowFISH, are powerful, high-throughput technologies to assess the activity of CREs [155,156]. Perturbation-based assays, including CRISPR interference/activation (CRISPRi/a) screens coupled with readout like FlowFISH or single-cell RNA-seq, directly link the disruption or activation of specific genomic loci to changes in target gene expression, enabling the functional mapping of regulatory networks [157,158,159,160]. Cryo-electron microscopy (Cryo-EM) provides high-resolution views of detection of colocalizing and chromatin architecture at CREs [161]. Furthermore, in vivo validation remains crucial, with methods such as transgenic mouse enhancer assays (e.g., enSERT) providing physiological context by testing candidate sequences in a developing organism [162].

## 3. *Cis*-Regulatory Elements as Key Modulators of Metabolic Reprogramming in HCC

### 3.1. CREs in the Regulation of Glucose Metabolism

**Figure 3:**
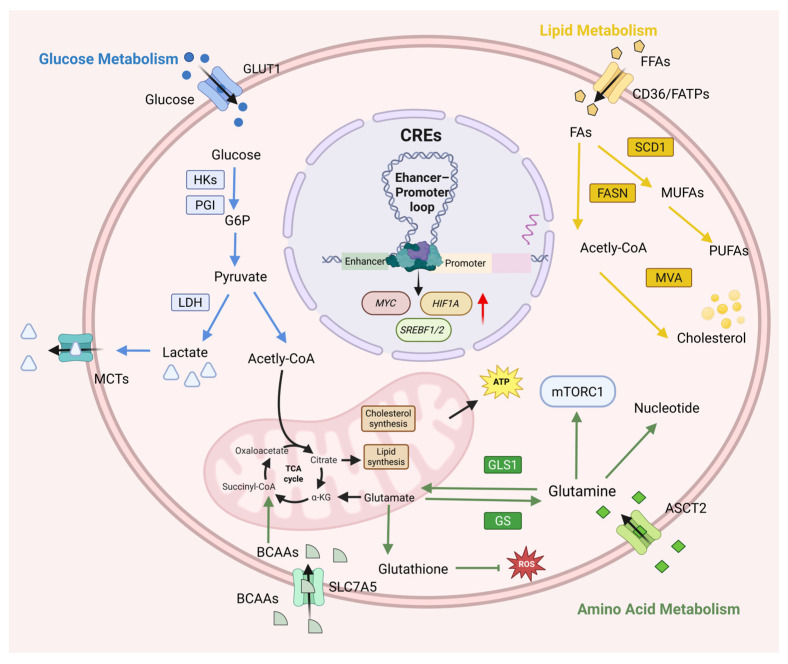
CREs as central modulators of tumor metabolism in HCC. This schematic illustrates how CREs, depicted here within an enhancer–promoter loop, orchestrate the reprogramming of key metabolic pathways in HCC. By integrating upstream signals from core oncogenic drivers (e.g., MYC, HIF-1α) and metabolic transcription factors (e.g., SREBP1/2), CREs coordinate the upregulation of genes involved in glycolysis (e.g., *GLUT1*, *LDHA*), lipogenesis (e.g., *FASN*, *SCD1*), and glutaminolysis (e.g., *GLS1*). This CRE-mediated metabolic rewiring sustains HCC progression by fueling rapid proliferation, biomass accumulation, and adaptation to the tumor microenvironment.

Glucose metabolism is central to energy production and biosynthesis in HCC. This reprogrammed metabolic process enhances glucose uptake and promotes glycolysis, enabling cancer cells to sustain rapid proliferation and survival within the challenging tumor microenvironment (Figure 3). The rewiring of glucose metabolism in HCC involves key pathways including glycolysis, the pentose phosphate pathway (PPP), gluconeogenesis and the tricarboxylic acid (TCA) cycle [163]. These changes are driven by altered expression of key metabolic enzymes. For instance, upregulation of the glucose transporter GLUT1 and hexokinase 2 (HK2) enhances glucose uptake and phosphorylation [164,165]. Preferential expression of the M2 isoform of pyruvate kinase promotes pyruvate accumulation. Overexpression of LDHA drives lactate production, reinforcing the Warburg effect [166]. Furthermore, increased activity of PPP enzymes like glucose-6-phosphate dehydrogenase and transketolase provides ribose-5-phosphate for biosynthesis and NADPH for redox balance [167]. This metabolic reprogramming is epigenetically regulated through *cis*-regulatory elements, including promoter methylation and enhancer activity, forming the core of glucose metabolic control in HCC.

DNA methylation within promoter regions is a pivotal epigenetic mechanism that regulates the expression of key enzymes in the glycolytic pathway, directly influencing the glycolytic phenotype of tumor cells. On one hand, aberrant hypermethylation of promoter regions tends to silence metabolic suppressors, thereby indirectly promoting glycolysis. For instance, hypermethylation of the *C1R* promoter leads to its downregulation, which subsequently activates the HIF-1α signaling pathway and drives glycolysis in HCC [168]. The silencing of *FBP1*, a rate-limiting gluconeogenic enzyme, via promoter hypermethylation, alters glucose metabolism and contributes to HCC progression [169,170]. Similarly, hypermethylation of the CpG island in the promoter of Derlin-3 (*DERL3*), a protein responsible for degrading GLUT1, results in GLUT1 stabilization and enhanced aerobic glycolysis [171]. On the other hand, promoter hypomethylation commonly drives the aberrant overexpression of critical glycolytic enzyme genes. In HCC, hypomethylation of *HK2* gene promoter directly promotes its transcription, increasing glycolytic flux [172,173]. The dynamic nature of this regulation is further highlighted by the role of demethylation enzymes. TET3 can also promote hepatic glucose production by depositing 5hmC marks on the promoter of gluconeogenic genes *PCK1* and *G6PC*, thereby inducing their expression [174]. Collectively, promoter DNA methylation finely tunes the expression of multiple key nodes in the glucose pathway, constituting a critical layer of epigenetic regulation in tumor metabolic reprogramming.

Enhancers and SEs critically regulate glucose metabolism by modulating the expression of key metabolic genes. The activation of oncogenes linked to these elements, such as MYC and SRC, leads to the transcriptional upregulation of a suite of genes involved in aerobic glycolysis, thereby reprogramming cellular glucose metabolic patterns [175,176]. A central mechanism involves E-boxes, which are essential *cis*-regulatory hubs for MYC-driven transcription. For instance, c-Myc directly binds to E-boxes in the promoters of key glycolytic genes like *GLUT1*, *HK2*, *ENO1*, *PKM2* and *LDHA*, initiating their expression to promote the Warburg effect, characterized by enhanced glucose uptake, aerobic glycolysis and lactate production [175,177]. This transcriptional program is often amplified by SEs. For example, the long non-coding RNA *CCAT1*, which functions as an enhancer RNA (eRNA), can modulate c-Myc expression by binding to the oncogenic 8q24 SE locus, a region associated with poor prognosis in HCC [178]. Beyond MYC, SE-associated RNAs like *HCCL5* enhance HCC invasiveness by indirectly regulating the expression of genes involved in glycolysis [179].

In the tumor microenvironment, rapidly proliferating cancer cells face the dual challenge of hypoxia and heightened energy demands. To adapt and survive, they activate specific transcriptional programs that reprogram glucose metabolism. A central regulator of this response is hypoxia-inducible factor 1 (HIF-1), which binds to hypoxia-responsive elements (HREs) in key metabolic genes and initiates their transcription [180]. This includes upregulating glucose transporters like *GLUT1* and glycolytic enzymes such as *PFK*, *HK*, and *LDH*, thereby shifting cellular metabolism from oxidative phosphorylation to enhanced glycolysis to support the Warburg effect [181,182]. Notably, a positive feedback loop exists, as LDHA has been shown to enhance HIF-1α stability by inhibiting its hydroxylation and proteasomal degradation, further amplifying glycolytic gene expression [182,183]. Beyond hypoxia, high extracellular glucose levels drive metabolic adaptation through carbohydrate response elements (ChoREs). These elements mediate the activity of the transcription factor ChREBP, which activates genes like *PKM2*, *GLUT1*, and *LDHA* [184].The high metabolic rate of tumor cells also generates substantial oxidative stress [185]. To counteract this, antioxidant response elements (AREs) and the transcription factor NRF2 play a pivotal role [186,187]. NRF2 orchestrates a metabolic shift by modulating genes involved in the pentose phosphate pathway and glycolysis, providing the redox balance and biosynthetic precursors necessary for tumor survival under stress [188,189].

### 3.2. CREs in the Regulation of Lipid Metabolism

The reprogramming of lipid metabolism in HCC is characterized by constitutive activation of de novo lipogenesis and systemic suppression of fatty acid β-oxidation, establishing a dysregulated state that fuels tumor progression [190]. This shift involves a coordinated network of processes, including fatty acid (FA) transport, de novo synthesis, complex lipid assembly, lipid droplet storage, and β-oxidation [191]. Lipids serve as critical energy stores and structural components for membrane biogenesis during rapid tumor proliferation. Notably, HCC cells intrinsically upregulate lipogenic pathways even under lipid-rich conditions via key enzymes such as ACC, FASN, and acyl-CoA synthetases [192]. Importantly, this metabolic phenotype is actively driven by a precise transcriptional program orchestrated by core transcription factors and their CREs, which coordinately induce lipogenic enzymes and suppress catabolic genes to support tumor growth and survival (Figure 3).

Promoter hypermethylation serves as a repressive epigenetic mark that downregulates key lipid metabolism genes. Environmental and metabolic factors initiate this process by modulating DNA methyltransferase (*DNMT*) expression [193]. For instance, high-fat diet (HFD)-induced upregulation of methyltransferases can promote hypermethylation of the *Klb* promoter and suppress its expression, contributing to hepatic steatosis [194]. Elevated glucose levels may also drive promoter hypermethylation via intermediates like 25-hydroxycholesterol (25HC), facilitating lipid accumulation through the epigenetic silencing of genes involved in cholesterol efflux or fatty acid β-oxidation [195]. Notably, the regulatory role of promoter methylation is dynamic and context-dependent. A key example is the developmental-stage-specific regulation of the *Gpam* gene. In the neonatal liver, DNA methylation at the *Gpam* promoter inhibits recruitment of the lipogenic transcription factor SREBP-1c, whereas in the adult liver, reduced methylation permits an active chromatin conformation and *SREBP-1c*-mediated transcriptional activation [193].

Sterol regulatory elements (SREs) are primarily bound by sterol-regulatory-element-binding proteins such as SREBP-1c and SREBP-2, to transcriptionally regulate lipid metabolism genes including *ACLY*, *ACC*, *FASN*, and *SCD1*, which are essential for fatty acid and triglyceride synthesis in proliferating tumor cells [196]. Consistent with this role, inhibition of the SREBP pathway has been shown to effectively reduce lipid accumulation in hepatocytes [197]. Moreover, SREBF2 and its downstream cholesterol synthesis genes such as *HMGCR* and *HMGCS1* boost cholesterol production critical for sorafenib resistance in HCC [198]. PPAR response elements (PPREs) are bound by peroxisome proliferator-activated receptors such as PPARα, PPARγ, and PPARδ to orchestrate transcriptional regulation of lipid metabolism in HCC [199]. The PPARγ-PPRE axis primarily promotes lipid accumulation by upregulating key lipogenic genes such as *FASN*, *ACACA*, and *SCD1*, thereby driving *de novo* lipogenesis and forming a positive feedback loop with *SREBP-1c* to enhance fatty acid and triglyceride synthesis [200]. Similarly, LXR response elements (LXREs) are bound by liver X receptors like LXRα/β to regulate lipid metabolism in HCC [201]. Under hypoxia, factors such as HIF-2α, whose expression can also be modulated by enhancer activity, participate in regulating key enzymes of fatty acid β-oxidation, including *CPT-1* [202]. Furthermore, SE-driven fatty-acid-synthesis-related lncRNA (*FASRL*) binds to ACC1, a rate-limiting enzyme in fatty acid synthesis, and inhibits its phosphorylation, thereby promoting fatty acid synthesis [14].

### 3.3. CREs in the Regulation of Amino Acid Metabolism

Amino acid metabolic reprogramming represents a central adaptive mechanism in HCC, characterized by a strong dependency on specific amino acids and profound alterations in metabolic pathways. HCC cells exhibit marked upregulation of glutaminolysis, with increased expression of glutamine transporter SLC1A5/ASCT2 and the enzyme GLS. This drives substantial glutamine uptake and catabolism to supply α-ketoglutarate (α-KG) for TCA cycle anaplerosis, while simultaneously supporting nucleotide synthesis, glutathione production, and NADPH generation to maintain redox homeostasis [203,204]. Branched-chain amino acid (BCAA) metabolism is also reprogrammed, as elevated BCAT1/BCAT2 expression promotes BCAA catabolism to generate branched-chain acyl-CoAs and α-KG, thereby fueling mTORC1 signaling and contributing to epigenetic regulation [205,206]. Furthermore, the serine/one-carbon metabolism pathway involving PHGDH, PSAT1, PSPH, and SHMT2 is strongly activated to provide precursors for nucleotide synthesis and methylation reactions, while upregulation of SLC7A11 enhances cystine uptake to bolster antioxidant defense [207]. This comprehensive reprogramming is orchestrated by a hierarchical network of CREs that integrates diverse oncogenic, metabolic, and differentiation signals (Figure 3).

Promoter hypermethylation serves as a pivotal epigenetic mechanism disrupting the expression of genes involved in amino acid transport, catabolism, anabolism, and related signaling. A prominent example is Glutaminase 2 (*GLS2*), a p53 target gene commonly silenced via promoter hypermethylation and silenced in human liver cancers [208]. Additionally, key urea cycle enzymes *CPS1* and *OTC* are frequently inactivated through promoter hypermethylation in HCC [209,210]. Hypermethylation of the *ASNS* promoter in liver cancer sensitizes cells to asparaginase treatment, which depletes plasma asparagine [211].

The oncogenic transcription factor MYC orchestrates glutamine and serine/glycine metabolic reprogramming by binding to E-box motifs within enhancers and promoters. This activity is amplified at SE hubs densely occupied by MYC, which co-regulate critical metabolic enzymes such as *GLS, SLC1A5, BCAT1,* and *SHMT2* to potentiate anabolic flux, thereby enforcing cellular dependence on glutamine as a primary carbon and nitrogen source [212,213]. This transcriptional control is further refined by other factors. For instance, HSF1 stimulates P300-mediated SE activity to facilitate the expression of *LINC00857*, contributing to SLC1A5-mediated glutamine transport [214]. Additionally, MYC upregulates enzymes like serine hydroxymethyltransferase and phosphoglycerate dehydrogenase, diverting glycolytic flux toward *de novo* synthesis of serine and glycine to supply one-carbon units essential for nucleotide biosynthesis and methylation reactions [215,216,217]. Beyond MYC, NRF2-associated SEs strongly activate *SLC7A11* and *GCLC* through antioxidant response elements (AREs), enhancing cystine dependency and glutathione synthesis in *NRF2*-mutant or NRF2-activated HCC subtypes [218]. In Wnt-activated HCC, the β-catenin/TCF4 complex binds to enhancers of the *GLUL* gene, establishing a metabolically flexible “glutamine cycle” that allows dynamic balance between glutamine synthesis and catabolism in response to microenvironmental fluctuations [219,220].

Under hypoxic conditions, stabilized HIF-1α/2α binds to HREs in target genes to modulate amino acid metabolism. For example, HIF-2α induces specific *GLS1* isoforms to sustain glutaminolysis and support cell survival under low oxygen [221]. HIF-1α/2α also activates *SLC1A5*, *GLS* and *SLC7A11* to enhance glutamine dependency, cystine uptake, and antioxidant defense, enabling HCC cells to maintain amino acid utilization and redox balance in hypoxic microenvironments [222]. In specific contexts such as hepatic stellate cells during fibrosis and NAFLD-associated HCC development, HIF-2α further promotes disease progression by enhancing glutamine catabolism [221].

Under metabolic stress such as nutrient deprivation, the integrated stress response induces *ATF4*, which binds to CRE-like elements in enhancers and promoters of serine synthesis pathway genes such as *ASNS*, *PHGDH*, *PSAT1* and *PSPH*, and recruits the co-activator p300 to drive their expression [223]. This redirects glycolytic flux toward serine and glycine production, supporting HCC adaptation in nutrient-limited microenvironments [223]. Furthermore, the histone demethylase KDM2B, along with ATF4 and MYC, forms an interconnected network regulating enzymes of the serine–glycine–one-carbon (SGOC), glutamate, and glutathione (GSH) metabolic pathways [224].

## 4. Metabolic State Modulates CREs Activity

The epigenome functions not as a static blueprint, but as a metabolically responsive regulatory layer. Chromatin-modifying enzymes are obligate consumers of key metabolic intermediates, making CRE activities sensitive to shifts in the intracellular metabolite milieu. In HCC, where metabolic reprogramming is pervasive, this biochemical coupling becomes a pathogenic driver of oncogenic gene expression.

### 4.1. Substrate and Cofactor Availability of Chromatin Modifiers

**Figure 4:**
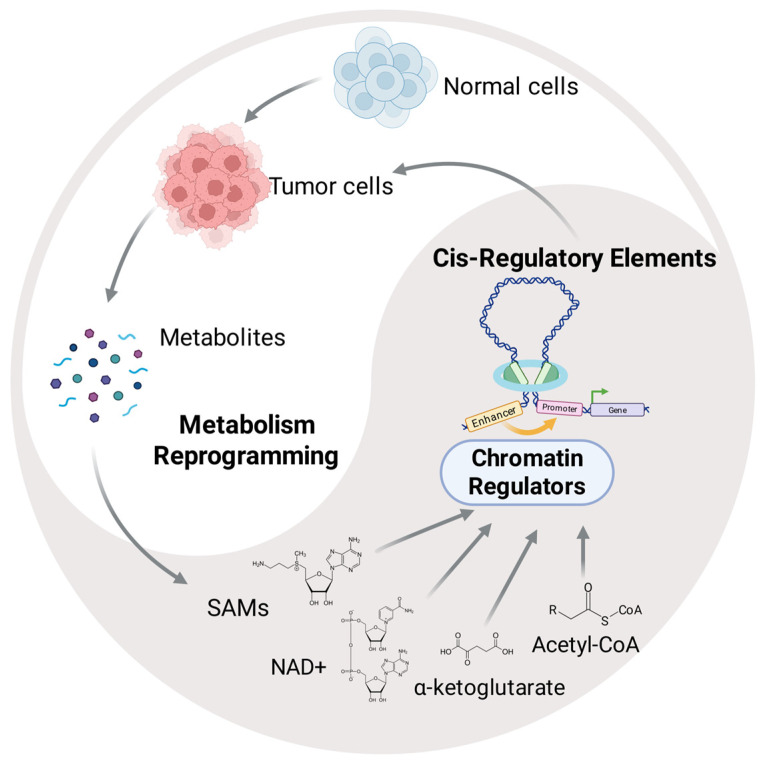
Metabolic regulation of CRE activity through chromatin modifier substrates and cofactors. The diagram illustrates a feed-forward loop linking metabolic reprogramming to the functional output of CREs in cancer. In HCC, metabolic reprogramming shifts the availability of key metabolites like acetyl-CoA, α-KG and SAM, altering chromatin regulator activity and consequently CRE functions such as enhancer activation, promoter accessibility, and 3D chromatin interactions. In turn, CRE-driven transcriptional programs further upregulate the same metabolic pathways, reinforcing a self-amplifying cycle. This reciprocal relationship establishes CREs as critical integration hubs where metabolic cues shape the epigenetic landscape, sustaining tumor growth, survival, and adaptation. Targeting these metabolite-enzyme-CRE axes may offer novel therapeutic strategies for HCC.

A profound bidirectional link exists between cellular metabolism and epigenetic regulation. Key intermediate metabolites directly act as substrates, cofactors, or competitive inhibitors for chromatin-modifying enzymes. This dynamic interaction modulates the histone and DNA modification landscape at CREs, thereby altering their transcriptional activity (Figure 4). This mechanism enables cells to rapidly adapt their gene expression programs in response to fluctuations in nutrient availability and energy status.

#### 4.1.1. S-Adenosylmethionine (SAM)

SAM serves as the principal methyl donor for epigenetic modifications, including those on DNA and histones. Consequently, cellular SAM levels, governed by the methionine and folate cycles, directly influence the activity of CREs. These metabolic pathways are intrinsically linked to cellular proliferation and differentiation, positioning SAM as a critical metabolic–epigenetic mediator [225]. Alterations in SAM levels exert profound and opposing effects on chromatin states. Elevated SAM promotes the deposition of repressive histone marks such as H3K27me3, leading to transcriptional silencing of tumor suppressor genes [226]. Conversely, SAM depletion induces global DNA and histone hypomethylation, which can activate normally silenced oncogenic elements [227]. This establishes a direct mechanistic link between SAM availability and CRE-mediated transcriptional activities in cancer. In HCC, dysregulation of one-carbon metabolism frequently disrupts SAM homeostasis, thereby perturbing histone methylation dynamics and CRE activity [228,229]. For instance, MAT1A overexpression in HepG2 and HuH7 cells elevated intracellular SAM levels, which resulted in suppressed proliferation and increased apoptosis [230]. Furthermore, elevated SAM contributes to the hypermethylation patterns characteristic of aggressive HCC subtypes, a phenomena supported by studies identifying aberrant methylation as central driver of hepatocarcinogenesis [231,232]. The overexpression of DNA methyltransferases like DNMT1 and DNMT3a in HCC tissues is often linked to this SAM-related metabolic dysregulation [233,234]. Thus, these findings underscore that one-carbon metabolism shapes the epigenetic landscape through SAM-dependent regulation of CREs. Targeting these metabolic pathways to restore SAM homeostasis emerges as a promising therapeutic strategy for mitigating the epigenetic dysregulation that drives HCC progression [235,236].

#### 4.1.2. Acetyl-CoA

Acetyl-CoA acts as a critical metabolic node connecting cellular metabolic flux to epigenetic regulation of gene transcription. As the essential acetyl donor for histone acetyltransferases (HATs), it directly links nutrient availability and metabolic state to the acylation status of chromatin at CREs, thereby modulating their activity [237]. Its cellular levels are governed by multiple pathways including glycolysis, fatty acid oxidation, and amino acid catabolism, enabling metabolic signals to directly influence chromatin accessibility and transcriptional programs in cancer [237]. The reprogrammed metabolism of cancer cells, notably the Warburg effect, significantly amplifies acetyl-CoA production [238]. This metabolic shift not only meets bioenergetic demands but also fuels epigenetic remodeling. In fatty liver disease, deregulated acetyl-CoA metabolism drives epigenome alterations that elevate carcinogenic risk [239]. Mechanistically, ATP-citrate lyase (ACLY) serves as a primary source of nuclear acetyl-CoA for histone acetylation, a pathway exploited by cancer cells to support proliferation [240]. Under metabolic stress (e.g., hypoxia), acetate from the tumor microenvironment can be converted to acetyl-CoA via ACSS2, providing an adaptive acetyl source to sustain tumor growth [241]. In HCC, acetyl-CoA accumulation has been shown to promote HCC metastasis via enhancing *CXCL1* expression, which in turn recruits tumor-associated neutrophils [17]. Given its role as a critical metabolic regulator of chromatin dynamics, acetyl-CoA represents a promising therapeutic target. Interventions aimed at normalizing its production or utilization may disrupt the metabolic–epigenetic circuit that sustains tumorigenesis and progression.

#### 4.1.3. NAD^+^

The availability of cofactors such as NAD^+^, NADP^+^, and various vitamin-derived molecules serves as a critical functional modulator for chromatin regulators, including histone acetyltransferases (HATs) and histone deacetylases (HDACs) [242]. Among these, NAD^+^ plays a particularly central role in oxidation–reduction (redox) reactions including glycolysis, the TCA cycle, OXPHOS, and fatty acid oxidation (FAO), directly influencing cell metabolism, genome stability, and histone modifications [243]. The intracellular NAD+/NADH ratio serves as a dynamic readout of cellular energy status, fluctuating in response to nutrient availability and metabolic flux, with a high NAD+/NADH ratio typically indicating an active metabolic state and a low ratio reflecting diminished energy production [244]. In parallel, multiple classes of transcriptional regulators have evolved to directly sense these redox changes, such as NAD+-dependent enzymes like Sirtuins (SIRT), NADH-sensitive transcriptional co-repressors including C-terminal binding protein (CtBP), NmrA-like redox sensor 1 (NMRAL1), and redox-responsive DNA-binding proteins like Redox regulator (Rex) [245,246,247]. SIRT catalyzes the removal of acyl groups from lysine residues, coupling the reaction to NAD^+^ hydrolysis and generating 2′-O-acyl-ADP ribose (OAADPR) and nicotinamide (NAM) [248]. Consequently, SIRT activity is finely tuned by cellular NAD^+^ and NAM levels, positioning them as metabolic sensors [249]. This metabolic–epigenetic coupling has significant functional consequences. For example, a metabolic shift from FAO to glycolysis decreases NAD^+^ levels, which inhibit SIRT1 activity, thereby impairing H4K16 deacetylation [249]. This demonstrates how metabolic reprogramming can directly rewrite the epigenetic state via NAD^+^. Similarly, the NAD^+^ salvage pathway enzymes NAMPT and NMNAT1 control gene expression in a SIRT1-dependent way. They regulate nuclear NAD^+^ concentration and SIRT1 deacetylase activity, thereby modulating H4K16ac levels at specific promoters. Intriguingly, SIRT1 can recruit NMNAT1 to target gene promoter regions, creating a microenvironment of high local NAD^+^ concentration to autoregulate its own activity [250]. Moreover, oncogenic signaling through the BRAF/ERK/STAT5 axis drives *NAMPT* transcription, resulting in elevated NAD^+^ levels and altered histone modification landscape, thereby promoting a more invasive cellular phenotype [251]. Thus, through cofactors like NAD^+^, cellular metabolism exerts direct and pleiotropic control over chromatin architecture and gene expression, with profound implications for cell fate and disease.

#### 4.1.4. α-Ketoglutarate

α-Ketoglutarate (α-KG), a key intermediate in the TCA cycle, serves as indispensable cofactor for a broad class of dioxygenase enzymes. This family includes histone demethylases such as the KDM family and TET DNA hydroxylases, which directly remodel chromatin structure by removing methyl marks from histone and DNA, thereby regulating gene transcription. Consequently, the cellular availability of α-KG directly modulates the activity of these chromatin modifiers, establishing a fundamental link between metabolic state and epigenetic control. In HCC, the functional balance of these dioxygenases is critically governed by the intracellular α-KG/succinate ratio. Oncogenic mutations in metabolic enzymes like succinate dehydrogenase (SDH) or fumarate hydratase (FH) lead to the pathological accumulation of succinate and fumarate. These metabolites act as competitive inhibitors of α-KG-dependent dioxygenases, resulting in a global hypermethylation of DNA and histones. This epigenetic silencing, particularly of tumor suppressor genes, drives cancer progression [252]. Conversely, sufficient α-KG levels support active demethylation, helping to maintain a more open and transcriptionally permissive chromatin state [253]. Thus, the regulation of α-KG metabolism represents a crucial interface between cellular bioenergetics and the dynamic control of gene expression through epigenetic modifications.

### 4.2. Oncometabolites Hijack CRE Regulation

Beyond the physiological fluctuations of metabolite pools, cancer cells exploit metabolic enzymes to generate oncometabolites, bioactive compounds that arise either from mutations in cancer-related genes or from hypoxia-induced enzyme promiscuity. These alterations cause normal metabolites to accumulate to abnormally high concentrations or lead to the production of noncanonical metabolites [254]. Notably, oncometabolites are frequently produced by mutations in nuclear-encoded TCA enzymes, including isocitrate dehydrogenase 1 and 2 (IDH1/2), succinate dehydrogenase (SDH), and fumarate hydratase (FH), all of which have been implicated in human cancers [255,256]. To date, four oncometabolites, namely 2-hydroxyglutarate, succinate, fumarate and lactate, have garnered substantial attention, though it is almost certain that additional oncometabolites will be identified in the future.

#### 4.2.1. 2-Hydroxyglutarate (2-HG)

The most prominent oncometabolite, 2-HG, is produced by mutated forms of IDH1 and IDH2. Both D-2-hydroxyglutarate (D-2-HG) and L-2-hydroxyglutarate (L-2-HG) enantiomers exist, with D-2HG arising from IDH1/2 mutations and L-2HG generated by lactate dehydrogenase and malate dehydrogenase under hypoxic conditions [257,258]. Due to its structural similarity to α-KG, 2-HG, particularly the D-enantiomer, competitively inhibits α-KG-dependent enzymes, including TETs, EGLNs, and KDMs. This inhibition disrupts the epigenetic regulation of DNA and histone methylation, culminating in a CpG island methylator phenotype (CIMP) characterized by hypermethylation and transcriptional silencing of hundreds of CREs, including tumor suppressor promoters [259]. IDH1/2 mutations occur in approximately 5–10% of HCC cases and are associated with a distinct epigenomic subtype with pervasive CRE silencing. Clinically, D-2-HG levels are significantly elevated in the plasma and liver of patients with biliary atresia and closely correlated with liver injuries and impaired liver regeneration [260]. Beyond its canonical epigenetic effects, L-2-HG has been shown to enhanced the lactylation modification of HIF-1α, thereby increasing resistance of cancer cells to ferroptosis and promoting proliferation, migration, and invasion [261].

#### 4.2.2. Succinate and Fumarate

Succinate and fumarate are intermediates of the TCA cycle. Under normal physiological conditions, these metabolites are rapidly converted into a tightly regulated sequence that drives ATP production. However, in cancer cells, this metabolic flux becomes disrupted, leading to their pathological accumulation [262]. This accumulation primarily results from defects in two key TCA cycle enzymes that function as tumor suppressors, including succinate dehydrogenase (SDH), which converts succinate to fumarate, and fumarate hydratase (FH), which converts fumarate to malate [252]. In HCC, multiple SDH subunits (SDHA/B/C/D) are significantly downregulated [263]. Notably, reduced expression of SDHB correlates with advanced tumor stage and poor survival outcomes in HCC patients [264]. Due to their structural similarity to α-KG, both succinate and fumarate act as competitive inhibitors of α-KG-dependent dioxygenases, including the Jumonji-C domain-containing histone demethylases (JHDMs) and the TET family [252]. This inhibition induces genome-wide alterations in histone and DNA methylation patterns, thereby reprogramming gene expression programs [265]. Interestingly, exogenous succinate has been shown to suppress HCC both in vitro and in vivo by acting as inhibitor of cholesterol biosynthesis, highlighting its potential therapeutic relevance [266].

#### 4.2.3. Lactate

In cancer, lactate functions as a critical energy substrate, metabolic metabolite, and signaling molecule that drives tumor progression through enhanced glycolytic flux and elevated cellular lactylation levels [267]. This metabolic reprogramming is fundamentally linked to the Warburg effect, which underscores the reliance of tumor cells on glycolysis and results in markedly increased lactate production [268]. Beyond its metabolic roles, lactate serves as an epigenetic modifier by promoting histone lactylation. Lactylome profiling of HBV-related HCC specimens has revealed high levels of lysine lactylation on enzymes involved in multiple metabolic pathways, including glycolysis, the TCA cycle, fatty acid metabolism, amino acid metabolism, and drug metabolism [269]. Specific histone lactylation sites have been implicated in HCC progression. For instance, acetylation of Lys 488 in the pyruvate dehydrogenase complex component X (PDHX), a modification commonly observed in HCC, disrupts pyruvate dehydrogenase complex (PDC) assembly, thereby contributing to lactate-driven epigenetic control of gene expression [270]. Additionally, lactylation of CENPA at K124 promotes its activation, leading to enhanced expression of target genes that drive HCC progression through cooperating with YY1 [271].

#### 4.2.4. Other Oncometabolites

Beyond the well-characterized oncometabolites discussed above, HCC features a broader repertoire of dysregulated metabolites that drive tumor progression through metabolic reprogramming, immune evasion, and epigenetic modifications.

Polyamines, including putrescine, spermidine, and spermine, are markedly elevated in HCC. These metabolites promote cell proliferation, chromatin remodeling, and an immunosuppressive tumor microenvironment by supporting mitochondrial function in tumor-associated macrophages [272]. Kynurenine, derived from tryptophan via the kynurenine pathway activated by enzymes such as IDO1 and TDO2, functions as a key oncometabolite. It binds the aryl hydrocarbon receptor to suppress anti-tumor T-cell responses, deplete tryptophan, and facilitate immune tolerance, with elevated levels correlating with poor prognosis [273]. Among amino acid derivatives, high arginine levels promote tumor formation through further metabolic reprogramming, including alterations in glucose, amino acid, nucleotide, and fatty acid metabolism [274]. Macrophage-derived itaconate, produced via IRG1/ACOD1, promotes HCC progression by epigenetically inducing CD8+ T-cell exhaustion, thereby enabling immune evasion [275]. Collectively, these metabolites underscore the reliance of HCC on altered metabolism and immune crosstalk, highlighting potential therapeutic targets such as pathway inhibitors or metabolite deprivation strategies.

### 4.3. Nutrition Reprograms CRE Activity in HCC

Nutrition serves as a direct modulator of epigenetic mechanisms through multiple pathways that control gene expression [276]. Overnutrition and associated metabolic alterations can trigger DNA and histone modifications via dysregulation of chromatin modifiers, resulting in aberrant transcriptional activity [277]. Nutritional factors encompass both dietary components and natural products, which collectively influence human health. HFD induces HCC by driving metabolic reprogramming that acts on the epigenetic machinery, particularly affecting CREs to alter gene expression [278,279]. HFD reduces hepatocyte differentiation and physiological output, perturbs hepatocyte functional balance, increases proliferation under stress, and directly primes future tumorigenesis [280]. Notably, HFD enriched in medium-chain fatty acids (MCFAs) from coconut oil (58% calories from fat) induces greater hepatic steatosis and triglyceride accumulation than a lard-based long-chain fatty acid (LCFA) HFD, likely due to upregulated lipogenic pathways, leading to pronounced weight gain and liver fat buildup [281].

Natural products originating from diverse sources, including plants, microorganisms, and marine sponges, exert anti-tumor effects by inducing specific epigenetic modifications in CREs, primarily through alterations in DNA methylation and histone modifications [282]. Several compounds demonstrate the capacity to directly target DNMTs. For instance, epigallocatechin-3-gallate (EGCG), a polyphenol found in green tea, inhibits DNMT activity, thereby contributing to the re-expression of silenced genes involved in cell cycle regulation and apoptosis in HCC models [283]. Similarly, epicatechin (EC) enhances DNA methylation at the GINS1 promoter, leading to reduced GINS1 expression and attenuation of liver cancer stem cell phenotypes and tumorigenesis [284]. Beyond DNMT targeting, other natural products exert anti-HCC effects through distinct mechanisms. Ginger, for example, has been shown to regulate lipid accumulation and ameliorates glucose uptake in HepG2 cells [285]. Ascorbic acid (AA) demonstrated anti-liver cancer efficacy both in vitro and in vivo, in a manner independent of stemness gene regulation [286].

### 4.4. Chromatin Architecture Dynamics Under Metabolic Stress

CRE activity does not operate in isolation; it is embedded within a three-dimensional chromatin architecture that constrains which enhancers can contact which promoters. Metabolic stress fundamentally reorganizes this architecture through the disruption of topological domain boundaries and the formation of new regulatory interactions. This dynamic has been characterized through chromatin structural and transcriptomic analyses in the context of HFD-induced obesity [24].

The most well-characterized mechanism involves metabolite-induced changes to CTCF binding, a key protein that maintains TAD boundaries. DNA methylation may play a broader role in regulating chromatin architecture by modulating CTCF occupancy [287]. Specifically, methyl donor availability directly controls the methylation status of CTCF-bound boundary elements, thereby linking dietary methionine and folate intake to 3D genome topology and subsequent oncogene activation [287]. In mice, fed-fast cycles dictate both CTCF expression and its chromatin association in the liver, with altered CTCF levels impacting hepatic transcription, energetics, and lipid metabolism [288]. Cohesin, the ring-shaped complex that mediates enhancer–promoter looping within TADs, is subject to post-translational regulation that responds to metabolic cues. The acetylation of cohesin’s SMC3 subunit is a dynamic process orchestrated by the acetyltransferase ESCO1 and the deacetylase HDAC8. This acetylation cycle critically controls the three-dimensional genome organization in human cells, linking metabolic status to chromatin looping dynamics [289].

Beyond boundary disruption, long-range promoter–enhancer interactions adapt to metabolic status through mechanisms that vary depending on the specific stress conditions. Adaptation occurs both through the activation of preformed chromatin loops and the *de novo* generation of entirely new loops [290]. This dynamic reorganization enables genes to be regulated via two distinct interaction mechanisms that respond differently to metabolic signals, providing flexibility in transcription adaption. Moreover, metabolic stress also induces phase separation of transcriptional condensates at super-enhancers. Intrinsically disordered regions of co-activators including p300, MED1 and BRD4 undergo liquid–liquid phase separation at H3K27ac-dense super-enhancers, concentrating the transcriptional machinery and amplifying CRE output [291,292]. Elevated acetyl-CoA under high-glucose conditions promotes H3K27ac spreading at super-enhancers, lowering the threshold for condensate formation and hypersensitizing oncogenic CREs to transcriptional activation [293,294]. This mechanism provides a rationale for why oncogenic super-enhancers in HCC are disproportionately sensitive to BET bromodomain inhibitors, which dissolve these condensates by displacing BRD4.

## 5. The Bidirectional Crosstalk Between Metabolism and CRE Activity

The relationship between metabolism and CRE activity is not unidirectional. While metabolic intermediates modulate chromatin-modifying enzyme activity to reshape the CRE landscape, the CREs themselves, through the genes, regulate feedback to control the production and consumption of those same metabolic intermediates. This bidirectional crosstalk constitutes a series of self-reinforcing circuits that, in HCC, become locked in oncogenic states.

### 5.1. Glucose–Acetyl-CoA–MYC Loop

The glucose-acetyl-CoA-MYC feedback loop represents a sophisticated regulatory circuit that drives HCC progression through interconnected metabolic and epigenetic mechanisms. At the molecular level, MYC functions as a super-enhancer-driven oncogene in HCC that simultaneously activates *GLUT1* to enhance glycolysis and upregulates *ACLY* to promote lipid biosynthesis by converting glycolytic intermediates into acetyl-CoA [295]. This metabolic coupling ensures a steady supply of acetyl-CoA for both energy production and epigenetic modifications, thereby sustaining proliferative signaling. MYC induces the production of mitochondrial acetyl-CoA, which can be subsequently converted to cytosolic acetyl-CoA via the citrate shuttle [296]. Elevated acetyl-CoA levels enhance histone acetylation in cancer cells, and given that histone acetylation facilitates transcriptional activation, the supply of acetyl-CoA as the substrate of histone acetyltransferases becomes critical for rapidly proliferating cancer cells [297].This creates a self-perpetuating cycle wherein MYC-driven glucose metabolism generates the acetyl-CoA required for its own transcriptional activation through chromatin modifications.

### 5.2. MAT1A Silencing–SAM Depletion–Hypomethylation Loop

The MAT1A silencing–SAM depletion–hypomethylation loop represents another self-reinforcing circuit with profound epigenetic consequences. In HCC, *MAT1A* undergoes promoter hypermethylation, leading to reduced expression and a MAT1A/MAT2A switch characterized by downregulation of *MAT1A* and upregulation of *MAT2A* [298]. This switch depletes hepatic S-adenosylmethionine (SAM), as MAT2A is less efficient at producing SAM [298]. Low SAM levels impairs DNMT activity, causing global DNA hypomethylation, a driver of genomic instability, aberrant gene expression, and oncogenic signaling [299]. Paradoxically, global hypomethylation exacerbates local hypomethylation at the *MAT1A* promoter, reinforcing its silencing and perpetuating SAM deficiency, proliferation, survival, and HCC progression [300]. The vicious cycle can be disrupted by SAM supplementation, which restores SAM pools, normalizes the MAT1A/MAT2A ratio, increases MAT1A expression in some models, and reduces tumor nodules and proliferation [19].

### 5.3. NAD^+^ Depletion–SIRT6 Loss–Warburg CRE Activation Loop

The NAD^+^ depletion–SIRT6 loss–Warburg CRE activation loop illustrates how cofactor availability directly modulates tumor suppressor function and metabolic reprogramming. NAD^+^ depletion reduces the activity of SIRT6, an NAD^+^-dependent deacetylase and tumor suppressor [301]. Under low-NAD^+^ conditions, SIRT6 fails to deacetylate H3K9 at promoters of HIF-1α target genes, resulting in increased H3K9ac and loss of HIF-1α co-repression. This upregulates glycolytic genes like *GLUT1* and *LDHA*, boosting glucose uptake, glycolysis, lactate production, and NADH accumulation [302]. The resulting NAD^+^ depletion further compromises SIRT6 activity, closing the loop to sustain proliferation and aggressiveness. Notably, NAD^+^ precursors such as nicotinamide mononucleotide or nicotinamide riboside restore NAD^+^ levels, reactivate SIRT6, suppress HIF-1α-driven glycolysis, and inhibit HCC growth, offering a potential therapeutic strategy to disrupt this oncogenic metabolic–epigenetic feedback loop [21].

## 6. Therapeutic Strategies for Targeting Oncogenic *Cis*-Regulatory Elements

CRE activity in HCC is governed by multiple interconnected regulatory layers, such as DNA methylation, histone modifications, chromatin accessibility, transcription factor binding, and cofactor recruitment. These layers are further organized into higher-order three-dimensional genomic structures, including TADs, enhancer–promoter loops, and phase-separated transcriptional condensates, through which CREs interact over long genomic distances. Critically, each regulatory layer represents a distinct therapeutic entry point for dismantling oncogenic CRE programs in HCC, offering opportunities for targeted intervention at multiple levels of epigenetic and chromatin organization.

### 6.1. Therapeutic Strategies Targeting DNA Methylation

DNA methyltransferase (DNMT) inhibitors: DNMT inhibitors such as decitabine and azacitidine have been developed to reverse aberrant DNA methylation patterns and reactivate silenced tumor suppressor genes [303,304]. These demethylating agents have received FDA approval for the treatment of hematological malignancies and are currently under investigation for solid tumors, including HCC [305]. Despite their promise, DNMT inhibitors as monotherapy have demonstrated limited efficacy in liver cancers. Studies using patient-derived cholangiocarcinoma cell lines revealed that decitabine and azacitidine exerted minimal effects on cancer cell proliferation when administrated alone [306]. This therapeutic limitation has promoted the development of combination strategies aimed at enhancing the anti-tumor potential of methylation inhibitors. Notably, combination approaches have proven more effective than single-agent regimens. For instance, PARP inhibitors act as sensitizers that synergistically enhance the anti-tumor effects of decitabine, demonstrating therapeutic benefit across multiple preclinical models, including patient-derived xenografts [306].

TERT promoter elements: Therapeutic strategies targeting TERT promoter elements primarily aim to disrupt transcription factor binding at *TERT* mutant sites. A promising strategy involves small molecules or oligonucleotides designed to block transcription factor binding at *TERT* promoter mutation sites. These mutations often generate *de novo* ETS/TCF transcription factor binding sites, leading to elevated *TERT* expression [106,307]. Inhibiting this interaction reduces *TERT* transcription and subsequently downregulates telomerase activity. Studies confirm that specific inhibitors or tailored oligonucleotides can effectively block transcription factor access and decrease *TERT* transcription in cancer cells, counteracting mutant-allele-driven tumor growth [308]. Another strategy employs epigenetic editing technologies such as the CRISPR-dCas9-KRAB system. This method enables precise targeting of *TERT* promoter mutations to introduce repressive chromatin marks, selectively silencing *TERT* expression. The fusion of the *KRAB* domain with catalytically inactive Cas9 (dCas9) recruits chromatin repressors to increase histone modifications that silence transcription [309,310]. This technology can specifically suppress transcriptionally active mutated *TERT* alleles while preserving normal alleles, minimizing off-target effects. Research has demonstrated that CRISPR-dCas9 systems significantly reduce *TERT* expression levels in various cancer cell types, including HCC [311,312]. Emerging evidence indicates that cancer cells with TERT promoter mutations exhibit increased sensitivity to polo-like kinase 1 (PLK1) inhibitors [313]. This principle is grounded on synthetic lethality, wherein impairing PLK1, essential for proper mitosis, disproportionately affects cells with elevated *TERT* levels, leading to cell death [313]. PLK1 inhibitors could thus serve as adjunct therapies for HCC patients with *TERT* promoter mutations [314]. Therefore, therapeutic strategies focusing on *TERT* promoter elements represent a promising direction for HCC treatment.

### 6.2. Therapeutic Strategies Targeting the Epigenetic Modification of CREs

Histone modifications define CRE identity and activity state. Consequently, drugs that inhibit or activate the enzymes responsible for writing or erasing these marks offer a direct mean to reprogram the oncogenic CRE landscape in HCC.

HDAC Inhibitors: HDAC inhibitors function by achieving site-specific histone hyperacetylation, thereby disrupting repressive chromatin architecture and facilitating transcriptional machinery recruitment to restore tumor suppressor gene expression [315]. To date, five HDAC inhibitors have received regulatory approved, namely vorinostat (SAHA), belinostat (PXD101), panobinostat (LBH589), romidepsin (FK-228), and tucidinostat (Chidamide) [316,317,318,319,320]. Currently, over 20 clinical studies are investigating these agents in refractory, advanced and recurrent solid tumors, including HCC, though no HDAC inhibitor has yet been approved specifically for HCC treatment [321,322]. Ongoing trials continue to evaluate HDAC inhibitors both as monotherapy and in combination with other modalities, including immunotherapy and targeted therapies [322]. Clinical experience to date suggests that while HDAC inhibitor monotherapy exhibits modest activity in HCC, combination strategies, particularly those incorporating immunotherapy or targeted agents like sorafenib, hold greater therapeutic promise.

EZH2 Inhibitors: EZH2, a core component of the Polycomb repressive complex 2 (PRC2), is overexpressed in approximately 30% of HCC cases and correlates with poor prognosis [323]. Tazemetostat (EPZ-6438), an FDA-approved EZH2 inhibitor, reduces H3K27me3 deposition and increases translocations in B cells with high AID activity or DNA repair deficiency [324]. In HCC models, tazemetostat synergizes with sorafenib and induces ferroptosis, enhancing sorafenib’s therapeutic effect on xenograft tumors [325]. Additionally, EZH2 inhibition reduces H3K27me3 at enhancers of immune checkpoint genes, suggesting a potential role in enhancing anti-tumor immunity [326].

HAT Activators and Inhibitors: Histone acetyltransferase (HAT) modulators represent an emerging class of epigenetic therapeutics. p300/CBP HAT inhibitors, such as A-485, have been shown to selectively inhibit proliferation across lineage-specific tumor types [327]. Beyond its anti-tumor effects, A-485 also inhibits lipogenesis in white adipose tissue and the liver, and decreases hepatic glucose production via preventing FOXO1 acetylation, thereby contributing to metabolic homeostasis [328]. In HCC, p300/CBP functions as critical epigenetic regulator of glycolysis-related metabolic enzymes, and B029-2, a p300/CBP inhibitor, has been proposed as a potential therapeutic strategy in this disease [329].

### 6.3. Therapeutic Strategies Targeting CRE Activation and Transcriptional Machinery

Directly targeting cis-regulatory elements and their associated transcriptional co-activators represents a promising strategy for disrupting oncogene expression in HCC.

BET Bromodomain Inhibitors: BET protein inhibitors represent the most clinically advanced approach for targeting super-enhancer function. BET proteins regulate multiple genes involved in cancer pathogenesis and have emerged as promising therapeutic targets, although clinical results indicate that their efficacy as single agents remains limited [330]. BET inhibitors, such as JQ1 and OTX015, selectively target BRD4 and other BET family proteins that are enriched at SEs [46,331]. By blocking BRD4’s recognition of acetylated histones, including H3K27ac, these agents disrupt SE-driven transcription, leading to rapid downregulation of key oncogenes and metabolic genes such as *MYC*, *CCND1*, *FASN* and *GLS* [46,332,333]. Preclinically, BET inhibitors suppress proliferation, induce apoptosis, reduce lipid accumulation, and inhibit tumor growth in HCC xenograft models [15,46,331,332,333,334,335,336]. Although no BET inhibitors have yet advanced to dedicated Phase II/III trials for HCC, Phase I studies in solid and hematologic malignancies have demonstrated tolerability and preliminary efficacy, supporting further investigation in HCC, particularly in combination with agents like sorafenib or immunotherapy.

CDK7 and CDK8/19 Inhibitors: Cyclin-dependent kinases (CDKs) involved in transcriptional regulation, including CDK8, CDK19, CDK7, CDK9 and CDK12/13, are critical for enhancer and SE function, primarily through their role in phosphorylating RNAP II and modulating co-activator complexes [337,338,339,340]. To date, however, no selective inhibitors targeting these CDKs have advanced to dedicated Phase III trials for HCC [341,342].

CBP/p300 Inhibitors: Targeting the histone acetyltransferases CBP and p300, which catalyze H3K27ac deposition at enhancers and SEs, represents an alternative strategy to disrupt CRE activity by promoting open chromatin and transcription factor recruitment [343]. Pharmacological inhibition of CBP/p300 using compounds such as CBP30 or MTL-CEBPA reduces H3K27ac levels, collapses SE activity, and downregulates associated oncogenes and metabolic genes in HCC [46,344]. Preclinical studies demonstrated that CBP/p300 epigenetically regulates the expression of glycolysis-related metabolic enzymes, and p300/CBP inhibitor B029-2 decreased glycolytic function and nucleotide synthesis [329]. Although these inhibitors remain in early-phase clinical testing for other cancer types, their potential relevance in HCC is underscored by the dependency of oncogenic and lipogenic SEs on H3K27ac. Combination strategies integrating CBP/p300 inhibitors with BET inhibitors or metabolic pathway inhibitors may offer enhanced therapeutic efficacy.

### 6.4. Therapeutic Strategies Targeting the 3D Genomic Architecture

The three-dimensional organization of CREs, including TAD boundaries, enhancer–promoter loops, and phase-separated condensates, is increasingly recognized as a therapeutic target in HCC. Dysregulation of this higher-order chromatin architecture represents a key mechanism driving oncogene activation and tumor progression in HCC.

CTCF, a master regulator of TAD boundaries and chromatin loops, has emerged as a promising therapeutic target. Strategies aimed at disrupting CTCF function have shown preclinical efficacy in multiple cancer models. For instance, Wen et al. constructed an artificial CTCF peptide (Decoy-CTCF) that significantly inhibited both proliferation and migration of ocular melanoma cells in vitro and in vivo by competing with endogenous CTCF for binding sites [345]. Beyond peptide-based approaches, small molecules have been identified that interfere with CTCF binding and chromatin organization. Tan et al. demonstrated that certain anthracycline derivatives, including aclarubicin and daunorubicin, can disrupt chromatin looping by directly interfering with CTCF occupancy at its cognate binding sites, thereby altering the spatial genome organization [346]. This disruption leads to changes in the regulation of associated genes, including the *MYC* locus, and correlates with distinct clinical outcomes in cancer patients [346]. These findings suggest that pharmacological modulation of 3D chromatin architecture may represent a viable therapeutic strategy, although its application in HCC remains to be explored.

### 6.5. Metabolic–Epigenetic Combination Therapies

The bidirectional crosstalk between metabolic reprogramming and CRE dysregulation in HCC provides a strong rationale for combination therapies that simultaneously target both axes. Metabolic intermediates, including acetyl-CoA, SAM, NAD+, and α-KG, serve as essential substrates and cofactors for chromatin-modifying enzymes that regulate CRE activity [295,296,297]. Disruption of these metabolic pathways can therefore indirectly modulate the epigenetic landscape at CREs, offering opportunities for synergistic therapeutic intervention.

SAM, the universal methyl donor for DNA and histone methyltransferases, is frequently depleted in HCC due to downregulation of methionine adenosyltransferases (MATs) [298,299,300]. SAM supplementation has been explored as a therapeutic strategy to restore normal methylation patterns at CREs [347]. Preclinical studies suggest that SAM can inhibit hepatocarcinogenesis by modulating epigenetic marks at tumor suppressor gene promoters, thereby reinforcing their expression [19,227]. Conversely, targeting the acetyl-CoA metabolic axis through inhibition of ACLY or modulation of mitochondrial acetyl-CoA production can alter histone acetylation at enhancers and SEs, which has potential to disrupt oncogenic transcriptional programs by limiting the substrate availability for histone acetyltransferases that activate CREs [296].

The combination of BET inhibitors with metabolic pathway inhibitors represents a particularly promising strategy. The dual PI3K/BRD4 inhibitor SF1126 exemplifies this approach, simultaneously targeting metabolic signaling through PI3K and epigenetic reading through BRD4, with demonstrated synergistic activity when combined with sorafenib in HCC [348,349]. Similarly, the combination of HDAC inhibitors with sorafenib has shown additive preclinical efficacy [317], and the integration of DNMT inhibitors with PARP inhibitors induces synergistic anti-tumor effects through complementary epigenetic mechanisms [306]. α-KG, a TCA cycle intermediate and essential cofactor for TET enzymes and JHDMs, has been shown to induce oxidative stress and mTOR inhibition, representing a distinct therapeutic strategy for liver cancer [20]. Collectively, these combination strategies leverage the inherent metabolic–epigenetic crosstalk in HCC to achieve more comprehensive disruption of oncogenic CRE networks than single-agent therapies, underscoring the importance of dual-axis targeting in future therapeutic development.

### 6.6. Current Challenges of CRE-Targeted Therapies

Despite the therapeutic promise of targeting oncogenic CREs, several resistance mechanisms and challenges continue to limit the translation of these strategies into durable clinical responses in HCC.

A primary obstacle is the development of adaptive resistance driven by epigenetic plasticity. Cancer cells possess an inherent capacity to compensate for the inhibition of one epigenetic pathway by activating alternative CRE regulatory mechanisms. For instance, resistance to BET inhibitors can emerge through the activation of alternative enhancers or transcription factor rewiring that bypasses BRD4 dependence, enabling sustained expression of oncogenic transcriptional programs [295]. Similarly, HDAC inhibitor resistance may develop through the upregulation of compensatory histone acetyltransferases or the activation of parallel signaling pathways that maintain CRE activity despite pharmacological inhibition [321,322].

The marked heterogeneity of CRE landscapes across HCC subtypes and individual tumors poses another significant challenge to effective targeting. Comprehensive epigenomic profiling has revealed substantial inter-patient variability in enhancer and SE activation patterns, suggesting that CRE-targeted therapies may require precise patient stratification based on robust epigenomic biomarkers [46,96]. The current lack of validated predictive biomarkers for patient selection remains a major obstacle to clinical translation, as it precludes the identification of those patients most likely to benefit from specific epigenetic interventions. Beyond heterogeneity, the functional redundancy inherent in CRE architecture may limit the efficacy of strategies targeting individual regulatory elements.

Significant pharmacological challenges persist in the clinical development of CRE-targeted therapies for HCC. Many epigenetic drugs, including HDAC and DNMT inhibitors, exhibit broad-spectrum activity that affects CREs genome-wide rather than selectively targeting oncogenic elements, leading to dose-limiting toxicities and off-target effects [318,320]. The development of more selective inhibitors, such as subtype-selective HDAC inhibitors like tucidinostat and isoform-specific BET degraders, aims to improve the therapeutic index by sparing normal CRE function while disrupting oncogenic transcriptional programs [319]. Emerging technologies such as CRISPR-based epigenome editing offer the potential for locus-specific CRE modulation, enabling precise activation or repression of individual regulatory elements. However, several obstacles must be addressed before clinical translation, including efficient and targeted delivery to HCC cells, minimization of off-target editing, and mitigation of immunogenicity concerns associated with CRISPR components [309,310,311].

The complex interplay between metabolic reprogramming and CRE regulation adds another layer of pharmacological complexity. Metabolic adaptation in HCC can dynamically alter the availability of epigenetic substrates and cofactors, potentially diminishing the efficacy of CRE-targeted therapies [297,302]. For instance, changes in SAM metabolism can shift DNA methylation patterns [298,299], while alterations in acetyl-CoA availability can modulate histone acetylation at enhancers [296], creating a constantly evolving epigenetic landscape that is difficult to target with static therapeutic approaches. This metabolic plasticity underscores the need for combination regimens that simultaneously target both metabolic and epigenetic dependencies.

In summary, current therapeutic strategies targeting CREs can be broadly categorized into two approaches, i.e., direct targeting of CREs or CRE-associated proteins, and indirect targeting based on the principle of synthetic lethality (Table 1). While significant progress has been made in preclinical models, the translation of these strategies to clinical practice requires overcoming interrelated challenges related to adaptive resistance, tumor heterogeneity, drug selectivity, and the dynamic nature of the metabolic–epigenetic interface. Further validation and refinement of these strategies, particularly through rational combination approaches, biomarker-guided patient selection, and the development of more selective epigenetic modulators, are expected to improve therapeutic efficacy and clinical outcomes in HCC.

## 7. Conclusions, Challenges and Future Perspectives

In summary, the intricate interplay between CREs and metabolic reprogramming represents a critical yet underexplored axis in HCC pathogenesis. Although extensive research has elucidated tumor-specific metabolic patterns across various cancers, the regulatory role of CREs in orchestrating these adaptations remains relatively understudied. Existing studies have primarily established correlative links between SEs and the expression of metabolic genes. However, mechanistic insights into how SEs specifically drive tumor metabolism, such as the recruitment of master transcription factors like MYC and HIF-1α to these elements and the precise processes governing enzyme and gene activation, remain limited. Moreover, enhancer–promoter interactions are likely to play an equally or more prominent role in enhancer selectivity and metabolic gene regulation. Yet, the differential contributions of various enhancer–promoter interaction models remain to be fully delineated. Furthermore, alterations in chromatin interactions, particularly enhancer–promoter looping in HCC and their adaptive responses to metabolic stress in precancerous states such as NAFLD, are incompletely characterized. A fundamental gap also exists in understanding how metabolite-induced global chromatin modifications occur at CREs. Similarly, the cooperative mechanisms between transcription factors and chromatin remodelers in sensing metabolic fluctuations remain poorly defined. Emerging questions further highlight the dynamic response of chromatin architecture to metabolic stress in chronic liver diseases, as well as the broader integration of metabolic signals beyond transcription. Importantly, metabolite fluctuations influence not only epigenetics but also RNA processing, translation, and protein degradation. How chromatin coordinates with these post-transcriptional steps of the central dogma to guide cell fate decisions remains largely unclear and will likely require holistic systems biology approaches. Addressing these challenges through advanced technologies, such as single-cell multi-omics, high-resolution chromatin conformation capture, and CRISPR-based perturbation of CREs, holds promise for uncovering novel therapeutic vulnerabilities. Ultimately, a deeper mechanistic understanding of the crosstalk between CRE and metabolism will illuminate new avenues for intervention in this aggressive malignancy and its metabolic precursors. 

## Figures and Tables

**Table 1:** Summary of inhibitors targeting CREs in HCC.

Target	Inhibitor	Target Model	Main Results	Clinical Phase	Ref
DNMT	Decitabine (5-aza-2’-deoxycytidine)	Human	Lower-dose decitabine achieved partial responses and disease stabilization in advanced HCC patients.	Phase I/II	[303]
DNMT	Decitabine	Cell line	Decitabine restored expression of epigenetically silenced tumor suppressor genes by demethylating promoter CREs.		[350]
DNMT	EGCG	Cell line/ Mouse	EGCG inhibited DNMT activity and reactivated methylation-silenced tumor suppressor genes in HCC models.		[282,283]
DNMT	Epicatechin	Cell line	Epicatechin attenuated liver cancer stemness through DNA methylation-mediated inactivation of GINS1/HRAS.		[284]
DNMT+PARP	DNMT inhibitor+ PARP inhibitor	Cell line/ Mouse	PARP inhibition augmented DNMT inhibitor efficacy by inducing senescence.		[306]
*TERT* promoter	BI2536; NMS-P937	Mouse	BI2536 or NMS-P937 could inhibit HCC tumor growth specifically in *TERT* promoter mutant (G228A) xenografts, but not in wild-type xenografts.		[314]
*TERT* promoter	CRISPR-dCas9-KRAB	Cell line	CRISPR-dCas9-KRAB selectively silenced mutant *TERT* allele by introducing repressive chromatin marks at *TERT* promoter.		[311,351]
HDAC	Romidepsin	Cell line/ Mouse	Romidepsin rendered liver cancer vulnerable to RTK targeting and enhances immunogenicity.		[316]
HDAC	Panobinostat	Mouse	Panobinostat showed additive preclinical efficacy when combined with sorafenib in HCC.		[317]
HDAC	Vorinostat	Human	Phase I study established pharmacokinetic and safety profiles in patients with advanced solid tumors and hepatic dysfunction.	Phase I	[318]
HDAC	Tucidinostat	Cell line/ Mouse	Tucidinostat showed therapeutic potential in cancer treatment.		[319]
HDAC	Belinostat	Human	This Phase I trial found that while liver dysfunction reduces belinostat clearance, the drug was well tolerated across all patient groups.	Phase I	[320]
EZH2	Tazemetostat	Cell line	The combination of the tazemetostat with sorafenib exhibits superior synergistic effects in anticancer therapy		[325]
BRD4	SF1126	Mouse	Treatment with SF1126 alone or in combination with sorafenib demonstrated significant antitumor activity in HCC	Phase I	[348,349]
BRD4	JQ1	Cell line	JQ1 reduced the expression of the SE-associated oncogenic transcripts and triggered large-scale transcriptional reprogramming genes in HCC cell lines.		[15,46,333]
BRD4	JQ1	Mouse	JQ1 inhibited tumor growth in HCC mouse model		[332]
BRD4	Birabresib (OTX-015)	Cell line	OTX-015 inhibited the proliferation of HCC cell lines.		[331]
BRD4	ABBV-075/ Mivebresib	Cell line	ABBV-075 inhibited the proliferation of HCC cell lines.		[334]
BRD4	AZD5153	Cell line	AZD5153 suppressed HCC growth by altering BRD4 landscape/transcriptome.		[335]
BRD4	OPT-0139	Cell line	OPT-0139 triggered apoptotic cell death and suppressed survival.		[336]
CDK1/2/7	Xylocydine	Mouse	Xylocydine inhibited the growth of HCC xenografts.		[341]
CDK7	THZ1	Cell line/ Mouse	SE-associated genes acquired in HCC cells were substantially reduced by THZ1		[46]
CDK7	3,3-difluorinated tetrahydropyridinol compound	Mouse	3,3-difluorinated tetrahydropyridinol compound suppressed tumor growth of HepG2 cell xenografts in nude mice.		[342]
EP300	CBP30	Cell line	CBP30 repressed the expression of the 13 SE- associated genes in HCC.		[46]
C/EBP	MTL-CEBPA	Human	MTL-CEBPA demonstrated an acceptable safety profile and potential synergistic efficacy with TKIs in HCC	Phase I	[344]
CTCF	Decoy-CTCF peptide	Cell line/ Mouse	Artificial CTCF peptide significantly inhibited proliferation and migration of cancer cells.		[345]
CTCF	Aclarubicin; Daunorubicin	Cell line	Anthracycline derivatives disrupted chromatin looping by interfering with CTCF binding and altering MYC locus regulation.		[346]
SPHK1	SKI-II	Cell line	SKI-II abolished the proliferation and colony formation of HCC cells		[46]
TET	Ascorbic acid	Cell line/Mouse	Ascorbic acid increased the concentration of H(2)O(2) and induced apoptosis in liver Cancer stem cells (CSCs).		[286]
TET	α-KG	Cell line	α-KG induced oxidative stress and mTOR inhibition.		[20]

## Data Availability

No new data were created or analyzed in this study. Data sharing is not applicable to this article.
